# Genomic epidemiology of a novel *Pandoraea pneumonica* group caused severe bloodstream infection in Hainan, China, 2021-2024

**DOI:** 10.3389/fcimb.2025.1560634

**Published:** 2025-04-28

**Authors:** Chong Chen, Min Wang, Tao Huang, Dong-liang Huang, Shuai Yu, Hui-min Zhao, Xiang-xiang Fu, Xin-xin Li, Hua Wu

**Affiliations:** ^1^ Department of Clinical Laboratory, Hainan General Hospital, Haikou, Hainan, China; ^2^ Department of Pharmacy, Hainan General Hospital (Hainan Medical University Hainan Hospital), Haikou, Hainan, China; ^3^ Emergency Department, Hainan Armed Police Force Hospital, Haikou, Hainan, China; ^4^ Department of Tropical Medicine, Hainan Hospital of People’s Liberation Army (PLA) General Hospital, Sanya, Hainan, China

**Keywords:** *Pandoraea pneumonica*, bloodstream infection, genomic epidemiology, whole-genome sequencing, pan-genome analysis

## Abstract

**Introduction:**

Rarely does *Pandoraea* occur in bloodstream infections (BSI), although it’s typically found in cystic fibrosis. This study aims to decipher the genetic map and obtain insights of clinical symptoms into Pandoraea from BSI patients.

**Methods:**

30 suspected BSI patients' diagnostic records and medical histories were recorded. *Pandoraea* spp. isolates were collected and subjected to antimicrobial susceptibility testing, Sanger sequencing and Whole-genome sequencing (WGS).

**Results:**

Of the 30 clinical cases, five (16.67%) ultimately died, whereas 25 (83.33%) are alive. 30 purified *Pandoraea* isolates showed high degree of MIC values to Meropenem, Amoxicillin and Potassium Clavulanate, Gentamicin, and Ceftazidime. Then, all isolates were identified as *P. pneumonica* based on the 16S rRNA-based phylogenetic analysis. Among 28 genomes of them, the average genome size and average GC contents were 5,397,568 bp, and 62.43%, respectively. However, WP1 displayed high similarity (90.6%) to reference *Pandoraea* sp. LMG 31114. Genetic differences between the tested isolates and LMG 31114 suggested that the outbreak’s causative pathogen could be a novel cluster of *P. pneumonica*. The genomes accumulated mutations at an estimated rate of 1.3 × 10^-7^ mutations/year/site. Moreover, 26 clinical isolates within the *P. pneumonica* cluster were formed in July 2014, revealing a tendency to develop regional endemic patterns.

**Conclusion:**

BSI caused by this novel cluster of *P. pneumonica* is linked to significant morbidity and mortality. Such cluster remains a critical public health challenge due to their regional epidemiological patterns and antibiotic treatment risk. This study contributed to the basis on pathogen identification, disease diagnosis, and BSI treatment.

## Introduction

Bloodstream infections (BSI), a growing public health burden and concern worldwide, are life-threatening human inflammation and dysfunction caused by a dysregulated host response to bacterial, fungal, and viral infections. Approximately 575,000–677,000 clinical cases/year and 79,000–94,000 deaths/year due to BSI have been reported in North America, whereas Europe experiences approximately 1,200,000 episodes and 157,000 deaths per year (Goto and Al-Hasan, 2013). The underlying cause of the infection may be used to distinguish or classify BSI cases. For instance, diagnostic codes for pneumonia, peritonitis, or urinary tract infections may be assigned to patients with BSI resulting from respiratory, gastrointestinal, or urinary tract sources of infection, respectively. Furthermore, BSI can go unnoticed as a cause of mortality, especially in individuals with end-stage renal or hepatic diseases, advanced cancer, or other terminal illnesses.

*Pandoraea* spp. serves as opportunistic pathogens in patients with cystic fibrosis (CF); however, their pathogenic mechanisms have not yet been systematically explored or understood. Epithelial cell invasion and increased levels of pro-inflammatory cytokines (interleukin [IL]-6 and IL-8) usually occur in these cases (Caraher et al., 2008; Green and Jones, 2018). Additionally, a CF individual with *Pandoraea* spp. was associated with BSI (Johnson et al., 2004) whereas six cases without CF were reported (Stryjewski et al., 2003; Falces-Romero et al., 2016; Xiao et al., 2019; Bodendoerfer et al., 2021; Gawalkar et al., 2021; Singh et al., 2021). Itoh et al. reported the first case of obstructive cholangitis related to bacteremia caused by *P. apista* which was identified using matrix-assisted laser desorption/ionization time-of-flight mass spectrometry and BLAST algorithms based on 16s ribosomal RNA sequence (Itoh et al., 2022). Another case of *Pandoraea pnomenusa* infection was recorded locally in Portugal revealed that the increased usage of carbapenems result in increased morbidity (Ramos Oliveira et al., 2023). Given the recent increase in the incidence rate of *Pandoraea* spp., it is imperative to comprehensively demonstrate its prevalence.

Most cases of CF caused by *Pandoraea* have been observed in Europe, America, and Australia, whereas in Asia, this type of case has rarely been reported (Lin et al., 2019; Singh et al., 2021). Patients with BSI caused by *Pandoraea* are even rarer. In addition to epidemiological data, the paucity of clinical reports indicate that the diagnosis of *Pandoraea* spp. is underestimated because of the challenges associated with standard diagnostic testing. For example, laboratory workers probably misidentify this microorganism as *Ralstonia* spp., *Stenotrophomonas* spp., or *Burkholderia* spp. when using only widely used assays, including the VITEK 2 automated microbiological system and standard phenotypic approaches (McMenamin et al., 2000; Bosshard et al., 2006). Furthermore, Sanger sequencing based on 16S ribosomal RNA and *gyrB* gene has low resolution for the classification of closely related *Pandoraea* spp. and poor traceability (Coenye and LiPuma, 2002; Pimentel and MacLeod, 2008). In contrast, high-throughput sequencing allows for classification and genetic evolutionary analysis based on bacterial whole genome. Besides, a whole map of core genes, antimicrobial resistance genes and virulence genes of infectious agents would be drawn (Shelenkov et al., 2020). It greatly benefits BSI patients in terms of an accurate diagnosis, preventing illness from worsening, shortening their hospital stay, and even improving survival rates.

In this study, the clinical presentations of 30 patients with suspected bacteremia or BSI were recorded. *Pandoraea* spp. were obtained from patient samples and subjected to whole-genome sequencing (WGS) and bioinformatic analyses to systematically analyze their epidemiology. This research offers a basic understanding of the relationship between *P. pneumonica* and clinical symptoms in patients as well as a theoretical direction for molecular diagnosis of this emerging pathogen.

## Materials and methods

### Clinical specimens

Based on hospital records, past patient data were retrieved. Sex, age, address, occupation, length of hospital stay, time required to isolate *Pandoraea* species, clinical symptoms at different times of the day, diagnostic evidence, use of antibiotics, use of antimicrobial drugs, underlying medical disorders, and patient outcomes were included in the data. The isolation of *Pandoraea* spp. from the blood was considered an infection based on the combined judgment of two infectious disease consultants.

Initial enriched cultures from positive blood culture mixtures were transferred to the enrichment culture plates using cell separation gradient solution on sheep blood agar and incubated aerobically at 37°C for 24–48 h. For morphological observations (the presence and kind of hemolysis, morphology, color, shape, size, and consistency) and primary identification, gram-negative bacteria were carefully sub-cultured on blood agar and MacConkey agar plates (Oxoid Ltd., Basingstoke, United Kingdom).

### Testing for antimicrobial susceptibility of *Pandoraea* isolates

The *Pandoraea* isolates were subjected for antimicrobial susceptibility testing, according to the detailed guidelines of the Clinical and Laboratory Standards Institute (CLSI). Mueller-Hinton Broth was applied for bacterial growth. The following 12 antibiotics were used in this study: Ceftazidime (CAZ), Piperacillin/Tazobactam (TZP), Amoxicillin/Clavulanate (AMC), Imipenem (IPM), Meropenem (MEM), Doxycycline (DOX), Chloramphenicol (CHL), Cefoperazone/sulbactam (SCF), Tigecycline (TGC), Ciprofloxacin (CIP), Trimethoprim-sulfamethoxazole (SXT) and Gentamicin (GEN). Since there were no criteria for antibiotic resistance of *Pandoraea* in CLSI at current, the *Burkholderia cepacian* was used as quality control organisms. Finally, the MIC values were determined visually after incubation for 18 hours.

### DNA extraction and genome sequencing

The whole genome of *Pandoraea* was extracted and purified using a 12224-250 DNeasy UltraClean Microbial Kit (Qiagen, Hilden, Germany) according to the manufacturer’s instructions. DNA quality was determined by 1.0% agarose gel electrophoresis, and the 260/280 absorbance ratios were determined using a NanoDrop2000 spectrophotometer (Thermo Fisher Scientific, Waltham, MA, USA).

For short-read sequencing, paired-end libraries were constructed following DNA fragmentation, end repair, and adaptor joining using Nextera XT library preparation kits (Illumina, San Diego, CA, USA). The libraries were cleaned using AMPure beads (Beckman Coulter Inc., Brea, CA, USA). Genomic libraries were assayed using a Flex Fluorometer Qubit4.0 and sequenced using a MiSeq sequencer (Illumina, San Diego, CA, USA).

For long-read sequencing, individual DNA libraries were prepared following DNA fragmentation, end repair, A-tailing, and adaptor joining using sequencing kit chemistries SQK-LSK109 (Oxford Nanopore Technologies, Oxford, United Kingdom). After library quantification using a Flex Fluorometer Qubit4.0, approximately 700 ng library was loaded onto an Oxford Nanopore MinION platform (Oxford Nanopore Technologies [ONT], Oxford, United Kingdom) for sequencing.

### Genome assembly and annotation

Quality control of Illumina sequencing reads and adapter trimming were performed using fastp (v0.23.2) with default parameters (Chen et al., 2018). *De novo* genome assembly based on clean reads from high-throughput sequencing was performed using SPAdes v.3.11 with default parameters (Prjibelski et al., 2020). To construct the complete genome, a hybrid assembly based on Illumina short reads and ONT long reads of WP1 was constructed using Unicycler 0.4.8 (Wick et al., 2017). Genome size and GC content were simultaneously measured and recorded. Subsequently, Prokka v1.1.3 was used to predict putative protein-coding genes with default parameters (Seemann, 2014).

### Comparative genomic analysis

Phylogenetic analysis based on 16S rDNA gene sequences was performed, and a neighbor-joining tree was visualized using MEGA X (Kumar et al., 2018). The average nucleotide identity (ANI) among all pairwise combinations of the whole genome of *Pandoraea* was computed using the BLASTN alignment tool in the Pyani package and visualized via an interactive heatmap (Pritchard et al., 2016). Core-genome single nucleotide polymorphism (SNP) analysis was performed using Parsnp software (V1.2) with the parameters “-c -x” (Kille et al., 2024). Core SNP positions were extracted using Harvesttools (Treangen et al., 2014) and then analyzed for recombination using the Gubbins software (Croucher et al., 2015). Tandem SNP loci after the removal of recombination were used to construct maximum likelihood trees using RaxML (Stamatakis et al., 2005), with the model set to GTRGAMMA and bootstrap of 1000 times.

### Pan-genome analysis and identification of virulence and antibiotic resistance factors

The pan-genome of *Pandoraea* spp. was performed using Prokka v1.1.3 tool generated feature files, and the families of homologous genes for *Pandoraea* spp. were assessed using Roary v3.11 with similarity cutoff value of 90.0% (Page et al., 2015). The virulence signatures and antimicrobial resistance genes were predicted from the representative sequences of pan-genome using the ABRicate software (v. 0.8.10) when both similarity and coverage were set to 60%.

### Inference of the evolutionary history of the novel *Pandoraea pneumonica* group

The best nucleotide substitution model general time-reversible (GTR) calculated using jModelTest 2.1.10 was selected for Bayesian inference using the core SNP alignment (Darriba et al., 2012). Molecular clock models (Strict and uncorrelated relaxed) and tree models (Constant Size, Exponential Growth, Expansion Growth, Bayesian SkyGrid, and Bayesian Skyline) were nested to test for convergence in the data set, and each model was run for 100,000,000 states at a 10,000 sampling frequency using BEAST software (v1.8.4). The Tracer program in BEAST was used to verify the convergence of every model combination (ESS of the tree model over 200). The maximum clade credibility (MCC) tree was generated using TreeAnnotator program in BEAST based on the best model (strict molecular clock and Bayesian Skyline tree model) and visualized using FigTree software (v1.4.3). The posterior mean values and 95% highest posterior density intervals of divergence time were estimated using 10 million Markov chain Monte Carlo generations, by sampling every 5000, and discarding the first 10% as burn-ins.

## Results and discussion

### Clinical symptoms of the infected patients

*Pandoraeae* spp. are usually isolated from environmental samples (soil, water, etc.) (Coenye et al., 2000). However, reports of BSI associated with comorbidities or complications are extremely rare. Herein, we described a large *P. pneumonica* outbreak in 30 patients. Between October 2021 and April 2024, 22 men and eight women were enrolled in this study (**Table 1**). One patient was 19 years old, seven were 35–49 years old, 15 were 50–69 years old, and seven were > 69 years old. In Hainan, 22 cases were found between May 15 and November 15 of the flood season, whereas eight cases were found at other times. During the flood season in extremely vulnerable communities in flooded areas, outbreaks of pathogenic infectious diseases, such as *E. coli* (Shah et al., 2016) and *Vibrio* spp (Esteves et al., 2015; Brumfield et al., 2021). increased. This might indicate a pathway of spread from the environment to humans. Itoh et al. have reported a similar clinical case (Itoh et al., 2022). The potential source of *P. apista* resulting in bacteremia was attributed to contamination of this pathogen from the environment during endoscopic ultrasound-guided hepaticogastrostomy surgery. Among the underlying medical disorders, 17 patients had gastrointestinal tract inflammation or peritoneal dialysis-associated peritonitis, 15 had high blood pressure, 10 were divided into a low-protein group, six had renal insufficiency, and four had anemia. The hypothesis that food-sourced *E. coli* cause BSI has been previously reported (Day et al., 2019). Further evidence is required to support the idea that eating food contaminated with pathogens can cause gastrointestinal tract inflammation and increase the risk of BSI caused by *Pandoraeae* spp. Additionally, four patients developed infections as a result of physical trauma, seven patients contracted infections after exposure to cold, and 17 patients had triggers that were unclear. In cases of severe trauma, 14.1% of patients with BSI had an initial serum procalcitonin level > 2 ng/mL (Rajkumari et al., 2013).

**Table 1:** Patient characteristics in an outbreak of *P. pneumonica* among BSI patients in Hainan during October 2021–April 2024.

Characteristics	Value
Sex:	
Male	22
Female	8
Age (year):	
0-19	1
20-34	0
35-49	7
50-69	15
>69	7
Occupation:	
Farmers	20
Other	10
Date of diagnosis:	
May 15 and November 15 of the flood season	22
Out of flood season	8
Underlying medical disorders:	
High blood pressure	15
Diabetes mellitus	2
Liver Insufficiency	2
Kidney Insufficiency	6
Tumor	2
Anemia	4
Low protein level	10
Triggers:	
No obvious triggers	17
Cold	7
Trauma	4
Exhaustion	1
After drinking alcohol	1
Clinical Symptoms:	
Fever	23
Cough	18
Chest tightness	11
Fatigue	11
Abdominal pain	8
Poor appetite	7
Nauseous	6
Vomiting	1
Shortness of breath	3
Antibiotic therapy:	
Yes	26
No	4
Diagnostic result:	
Bacteremia	17
Lung infections or pneumonia	19
Hypoproteinemia	10
Hypopotassaemia	12
Liver damage	4
Liver failure	4
Renal insufficiency	4
Kidney failure	2
Ending:	
Survival	25
Death	5

The assessment of clinical symptoms in patients with suspected bacteremia or BSI during this outbreak is not always straightforward. Twenty-three patients had fever, 11 had chest tightness, 11 had exhaustion, eight had abdominal pain, seven had poor appetite, six had nausea, and 3 had decreased consciousness during the infection period (**Table 1**). BSI is caused by a wide variety of pathogens but carries clinical syndromes with considerable overlap, including fever, chills, and changes in mental status (Martinez and Wolk, 2016). In addition to *Pandoraeae* spp., *Ralstonia pickettii*, *Escherichia coli*, *Burkholderia cepacian*, and *Leifsonia aquatica* was detected in the blood cultures of four, one, one and one patients, respectively. Additionally, 19 patients had potential pulmonary infection, among which five were validated to have co-infections (two with severe acute respiratory syndrome coronavirus 2, one with *Klebsiella pneumoniae*, and two with *P. aeruginosa*). Notably, one patient with acute diffuse peritonitis, one patient who had co-infection with *B. cepacian*, and three patients with pulmonary co-infections died (**Supplementary Table S1**). Even while this study was unable to provide direct evidence for the higher death rate among individuals with co-infections or co-morbidities, we must focus on the distinct variations among pathogens, co-infection complexity, surveillance data, and several factors linked to unfavorable consequences (such as disability and death) and therapeutic challenges. Quick and precise molecular testing will contribute to rapid precision therapy. This necessitates a methodological understanding of the entire pathogen genome sequence.

### Antibiotic susceptibility of *Pandoraea* isolates

Most of the patients survived with antibiotic treatments, but there were still four deaths who were treated with antibiotics. Therefore, we then tested for the antibiotic susceptibility profiles of these *Pandoraea* isolates. The MIC50, MIC90, MIC range and percent susceptibility for tested antibiotics are listed in **Table 2**. Referenced to the criteria of *Burkholderia cepacian* and the quality control organisms (Clinical and Laboratory Standards Institute (CLSI), 2025), the tested *Pandoraea* isolates showed high degree of MIC values to MEM (MIC50 and MIC90: ≧16 μg/mL), AMC (MIC50 and MIC90: ≧128/64 μg/mL), GEN (MIC50 and MIC90: ≧16 μg/mL), and CAZ (MIC50 and MIC90: ≧128 μg/mL). It was also reported that high frequency (76.7%, 23/30) of *Pandoraea* isolates exhibited resistance to MEM (Lin et al., 2019). In contrast, MIC50 and MIC90 values of DOX and TGC were at least 8-fold lower than MEM, AMC, GEN and CAZ. Collectively, these findings suggested that the *Pandoraea* isolates had potential resistance to various antibiotics. However, there is currently no criteria for the evaluation of MIC values in *Pandoraea*, we were unable to directly determine the resistance level of the isolates in this study. Further investigations are needed to understand the susceptibility patterns of *Pandoraea*, which would help clinicians choose effective antibiotics and inform public health strategies to combat resistance.

**Table 2:** MIC value and activity spectrum of 12 antibiotics against *P. pneumonica* clinical isolates.

Antibiotic	MIC (μg/mL)		
	MIC50	MIC90	Range
Ceftazidime (CAZ)	≧128 (17/28)	≧128 (17/28)	16~≧128
Piperacillin Sodium and Tazobactam Sodium (TZP)	8/4 (3/28)	≧16/4 (12/28)	0.125/4~≧16/4
Amoxicillin and Potassium Clavulanate (AMC)	≧128/64 (28/28)	≧128/64 (28/28)	≧128/64
Imipenem (IPM)	2 (21/28)	4 (5/28)	≦0.064~4
Meropenem (MEM)	≧16 (28/28)	≧16 (28/28)	≧16
Doxycycline (DOX)	1 (18/28)	2 (2/28)	≦0.125~16
Chloramphenicol (CHL)	16 (22/28)	32 (4/28)	8~32
Cefoperazone/sulbactam (SCF)	2/1 (21/28)	8/4 (1/28)	1/0.5~16/8
Tigecycline (TGC)	1 (21/28)	2 (3/28)	0.5~2
Ciprofloxacin (CIP)	4 (15/28)	8 (11/28)	2~16
Trimethoprim-sulfamethoxazole (SXT)	0.25/4.75 (23/28)	0.5/9.5 (5/28)	0.25/4.7~0.5/9.5
Gentamicin (GEN)	≧16 (28/28)	≧16 (28/28)	≧16

### Determination of the novel *Pandoraea pneumonica* group based on genome sequencing

**Figure 1:**
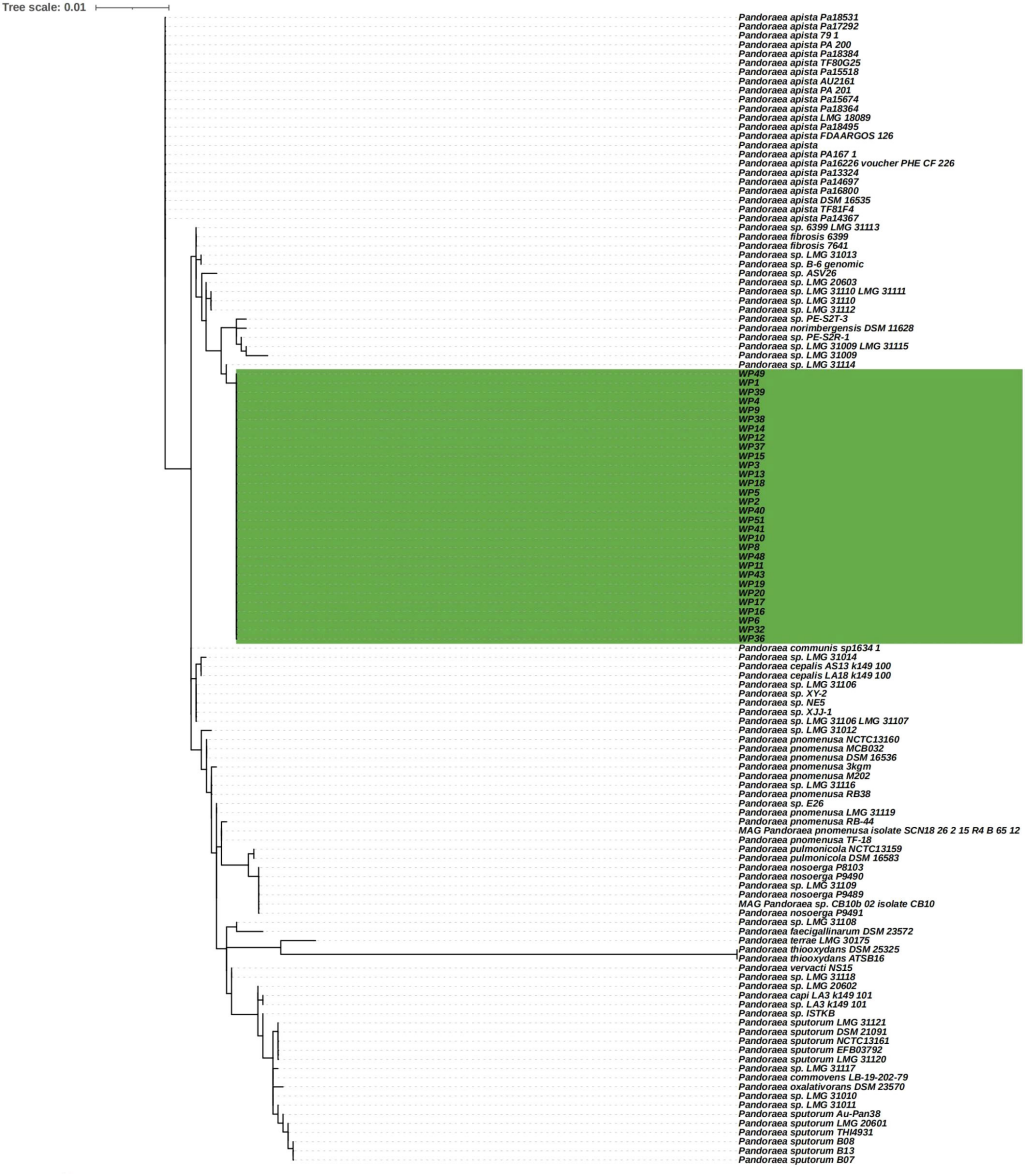
Phylogenetic tree based on 16S rDNA gene sequencing showing *P. pneumonica* isolates and other related reference strain from the NCBI database.

Whole blood samples were collected for the isolation of *Pandoraea* spp. and 30 isolates were used for further analysis. A phylogenetic tree based on 16S rDNA gene sequences was constructed using representative sequences of the genus *Pandoraea* and our isolates. All 28 isolates and *Pandoraea* sp. LMG 31114 were divided into the same large clusters (**Figure 1**). Thus, it has been suggested that they originated from a common, distant ancestor. *Pandoraea* sp. LMG 31114 was firstly isolated from CF patient in the United States in 2009 (Peeters et al., 2019).

The complete genome of pathogenic microorganisms are plastic on an evolutionary time scale. But high-throughput sequencing can be applied for research on overall structure, organization and function of them. The complete genome sequence of WP1 was obtained by Nanopore sequencing. The WP1 genome has one chromosome of 5,773,419 bp, containing 5,100 coding sequences, 12 rRNA genes, 68 tRNA genes, and one tmRNA. Subsequently, draft genomes of the remaining 27 isolates were sequenced (**Supplementary Table S2**). Among the genomes of the 28 *Pandoraea* isolates, the minimum, maximum, and average genome size was 4,442,224 bp, 5,826,184 bp, and 5,397,568 bp, respectively. The minimum, maximum, and, average GC contents were 62.11%, 62.51%, and 62.43%, respectively. To the best of our knowledge, only a few whole genome sequences of *P. pneumonica* have been reported. Peeters et al. demonstrated that P*. pneumonica* sp. nov. (type strain LMG 31114T = CCUG 73388T) from a patient with CF had a 5,845,078 bp nucleotide sequence with 62.5% GC content (Peeters et al., 2019). Their genome size and GC content were consistent with those of the 28 isolates in this study.

**Figure 2:**
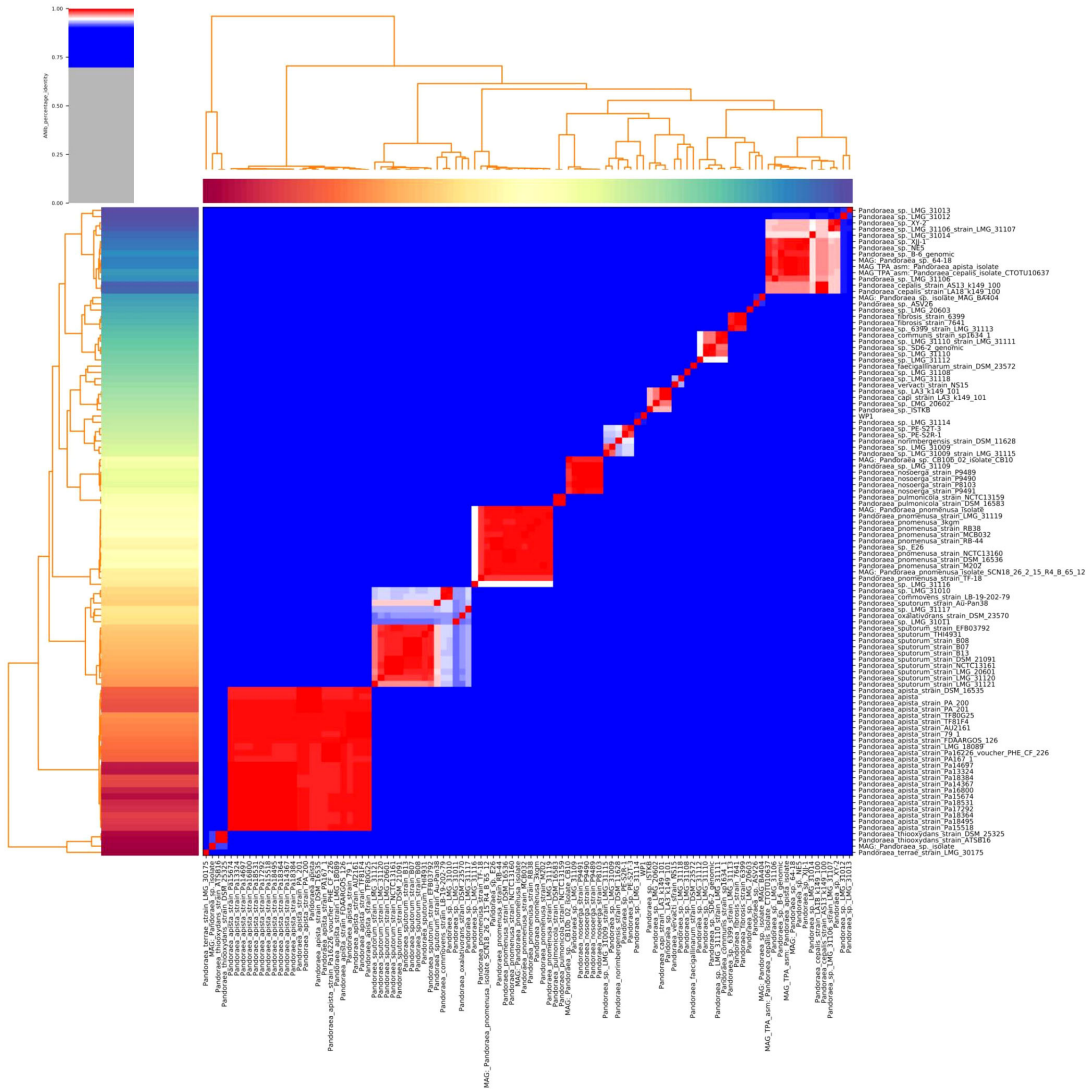
Heatmap of the average nucleotide identity (ANI) values for whole genomes, sequenced from related *Pandoraea* spp. in the NCBI database, including WP1 from this study. Values range from 0 (0% ANI) to 1 (100% ANI): gray represents 0% ANI.

Furthermore, the homology of the WP1 whole genome was analyzed via ANI analysis. As shown in **Figure 2**, WP1 displayed high similarity (90.6%) to *Pandoraea* sp. LMG 31114. The genus is a cohesive group that shares a high degree of nucleotide sequence similarity (> 85.0%), with two species belonging to the same species differing by < 5% from each other at the nucleotide level, according to ANI analysis (Goris et al., 2007; Richter and Rossello-Mora, 2009). Thus, we hypothesized that WP1 is a novel subspecies or even a novel member of a species similar to *P. pneumonica*.

The SNP classification strategy on the core genome (defined as orthologous sequences conserved in all aligned genomes) allows phylogenetic reconstruction, clade discrimination, and genetic distance calculation by SNP alignment, base-by-base comparison, and consideration of evolutionary models with masked recombination locations as well as mobile elements (Abdel-Glil et al., 2022). As shown in **Supplementary Figure S1**, 28 isolates clustered together, but diverged in LMG 31114. The inner genetic distance within the 28 tested isolates was < 100 bases, whereas the genetic distance within LMG 31114 was > 100,000 bases. This result further suggests that these isolates can be defined as a novel cluster of *P. pneumonica*.

### Potential virulence factors and antibiotic resistance genes

**Figure 3:**
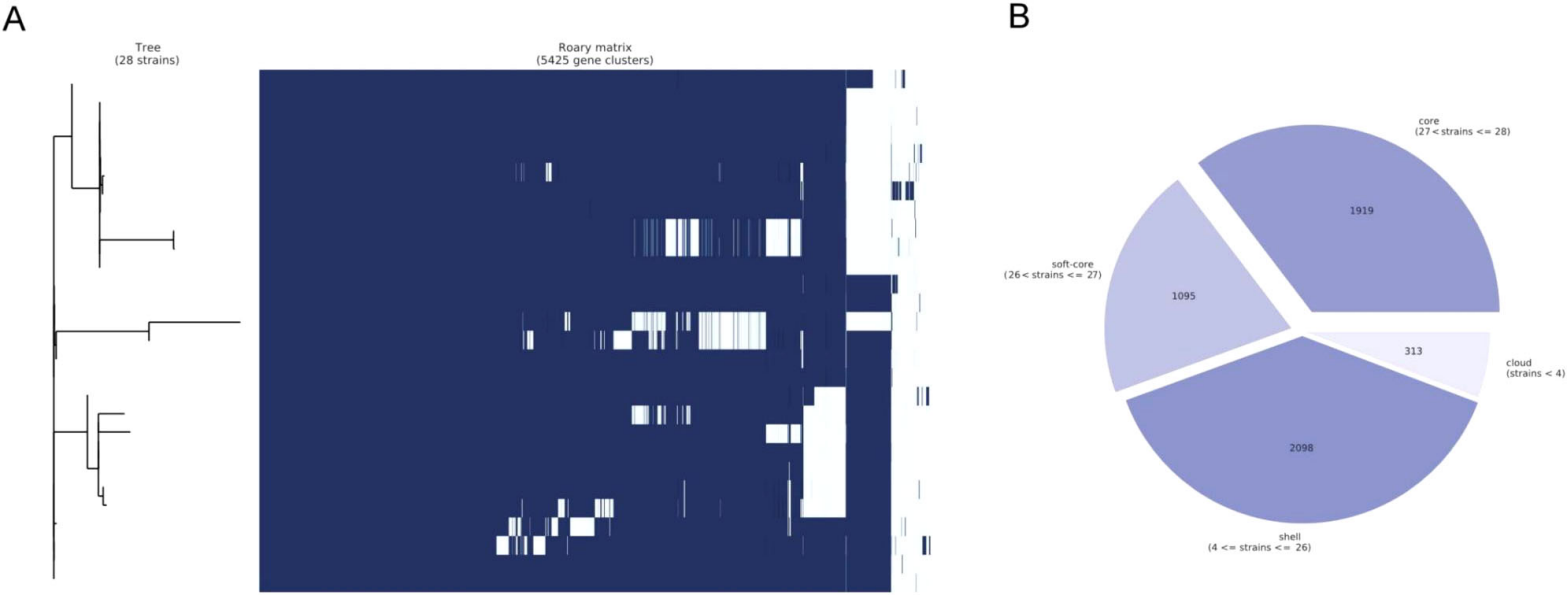
Pan-genome analysis. **(A)** Clustered heatmap based on the shared genes. Multiple sequence alignments were performed for each core gene in each isolate, and combined together to build the phylogenetic tree. **(B)** Statistical information on core and soft-core genes. During the statistical analysis, core genes were defined as genes present in 28 isolates, soft genes were defined as genes present in 27 isolates, shell genes were present in at least 4 isolates and no greater than 26 isolates, and cloud genes were present in less than 4 isolates.

The total number of pan genes in this new *P. pneumonica* cluster was 5425, of which the core and soft core genes were 1919 and 1095, respectively, accounting for 55% of the total number of genes (**Figures 3A, B**). In total, 46 potential virulence factor genes were predicted, of which 38 were core genes, including *cheY* with 99.24% coverage, *tufA 2* with 98.65% coverage, *tufA 1* with 98.65% coverage, *adeG* with 97.61% coverage, and *flgG* with 97.59% coverage (**Supplementary Table S3**). However, the similarity between these genes was < 85% compared to those reported in the Virulence Factor Database. *adeG*, which encodes the cation/multidrug efflux pump AdeG, serves as a biofilm-controlling response regulator related to biofilm formation (the major virulence factor of *Acinetobacter baumannii*) (Mea et al., 2021; Shan et al., 2022).

Seventeen potential resistance genes were identified, of which 10 were core genes, including *ceoB* with 99.42% coverage, *MexB* with 97.33% coverage, *MuxB* with 96.04% coverage, *MexA* with 84.72% coverage, and *OrpM* with 85.6% coverage (**Supplementary Table S4**). Except for *OXA-157* (85.31% coverage and 86.61% identity), the similarity between the remaining genes and drug resistance genes in the Comprehensive Antibiotic Resistance Database was < 85%. Combined with phenotype results of this study, we highlighted the potential relation between MEM and *OXA-157*. Previously, the influence of *OXA-151* to *OXA-159* on the MEM-resistant phenotype of *Pandoraea* spp. was observed by Schneider et al (Schneider and Bauernfeind, 2015). Thus, OXA-157, coding oxacillinases (β-lactamases of the molecular class D), may serve as a critical factor on MEM resistance. *MuxB* contributes to muxABC-opmB synthesis, a putative resistance nodulation cell division (RND)-type multidrug efflux pump system, in *Pseudomonas aeruginosa* (Lorusso et al., 2022). Three membrane-bound subunits (MexA, MexB, and OprM) anchor the inner and outer membranes associated with another RND system MexAB-OprM (Paulsen et al., 1996; Tseng et al., 1999; Akama et al., 2004). Based on the outcomes of gene disruption, such a mechanism might be considered to be essential for the resistance to CAZ and CIP in *P. pneumonica* (Li and Plésiat, 2016). Further research is required on additional resistance phenotypes and pathogenic pathways.

### Evolution of the novel *Pandoraea pneumonica* group

**Figure 4:**
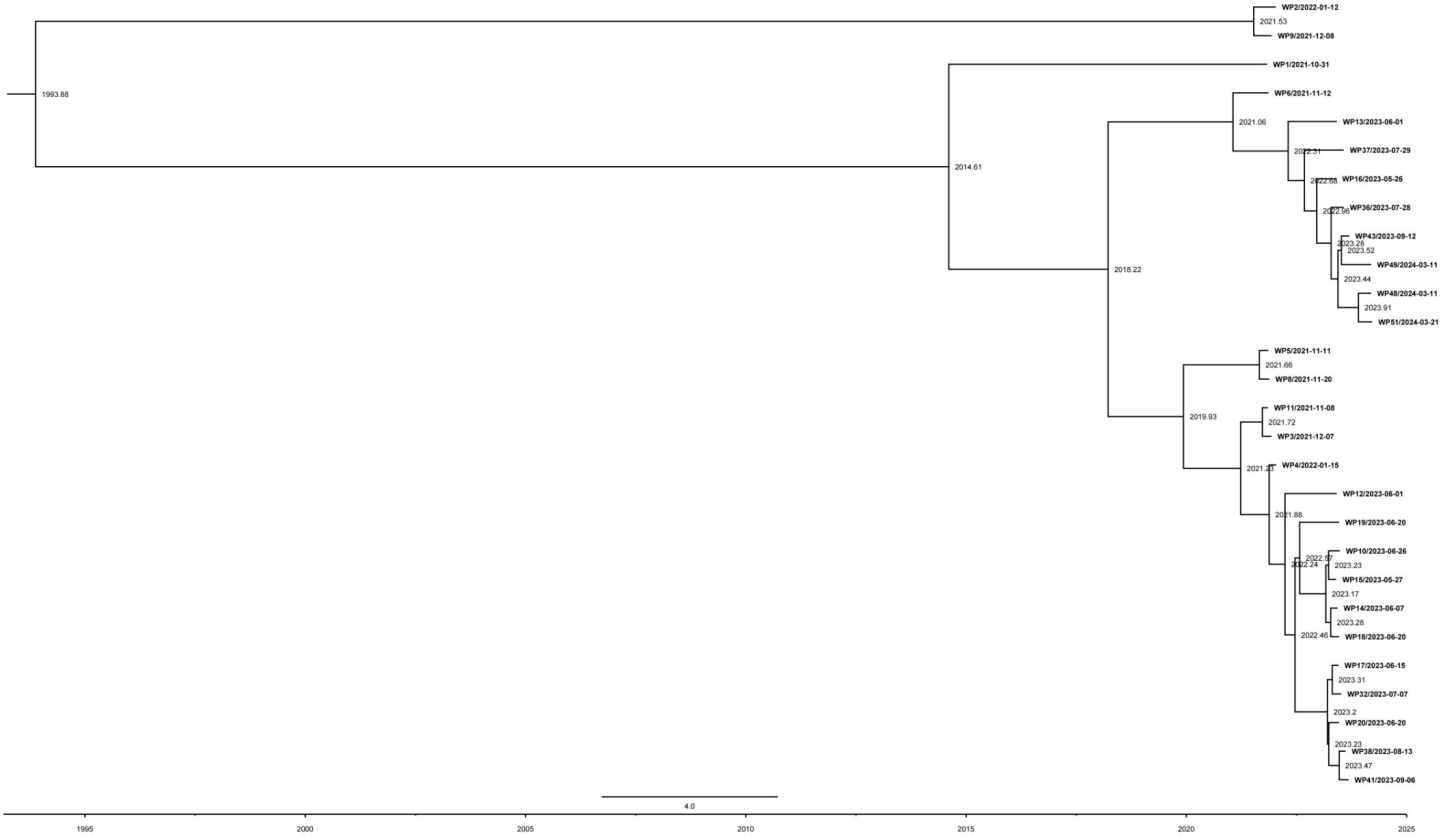
Evolutionary reconstruction of *P. pneumonica* isolates. A time-calibrated maximum clade credibility tree was inferred from core-genome single-nucleotide polymorphisms (SNPs) from *P. pneumonica* genomes. The X-axis represents the emergence time estimates.

The ML phylogeny was inputted into TempEST to examine the temporal signal of the dataset. A linear association between evolutionary distance and divergence time was observed. The genomes accumulated mutations at a rate of 1.3 × 10^-7^ substitutions per site annually, or roughly 0.27 SNPs (**Figure 4**). Based on root-to-tip divergence analysis, the most recent common ancestor of the *P. pneumonica* lineage was estimated to have emerged in 1993. The WP9 and WP2 clusters were formed between December 2021 and January 2022, whereas other distinct clinical isolates within the *P. pneumonica* cluster were formed at the earliest in July 2014. We also hypothesized that isolates with comparable geographic origins might carry a clonal link and develop regional endemic patterns. Overall, this *P. pneumonica* cluster contained few SNPs and was genetically homogeneous. These results indicate that all strains exhibited a high degree of relatedness and a close phylogenetic link. To the best of our knowledge, this is the first study to demonstrate the rate of mutations in the core genome of *P. pneumonica* based on the whole genome sequences acquired through high-throughput sequencing. Usually, a genome-wide mutation rate of approximately 10^-7^ mutations/year/site occur in *E. col*i (White et al., 2024), *Shigella* (Holt et al., 2013), and *Chlamydia psittaci* (White et al., 2022; Kasimov et al., 2023). Genetic epidemiology in this study revealed clusters of isolates, which may indicate that patients contracted the infection from a common source. This finding highlights the advantages of integrating epidemiological data from WGS into public health surveillance and research.

## Conclusion

Our study highlighted the benefits of bacterial WGS for precise isolate identification and epidemic classification. Based on these findings, including clinical symptoms and whole-genome bioinformatics analysis, we concluded that a novel cluster of *P. pneumonica* serves as a critical emerging pathogen in people with BSI. Comorbidities or complications make the patient’s condition more difficult. This outbreak report demonstrates the importance of the vigilance of microbiologists and doctors when novel members of pathogenic microorganisms are detected. Medical testing laboratories must develop and update cutting-edge molecular sequencing technologies. Additionally, a more comprehensive genomic epidemiology study is necessary to promote future diagnosis and therapy of this pathogen.

## Data Availability

The original contributions presented in the study are publicly available. This data can be found here: [https://www.ncbi.nlm.nih.gov/bioproject/?term=PRJNA1250782, https://www.ncbi.nlm.nih.gov/bioproject/?term=PRJNA1250798, https://www.ncbi.nlm.nih.gov/bioproject/?term=PRJNA1250801, https://www.ncbi.nlm.nih.gov/bioproject/?term=PRJNA1250804, https://www.ncbi.nlm.nih.gov/bioproject/?term=PRJNA1250810, https://www.ncbi.nlm.nih.gov/bioproject/?term=PRJNA1251137, https://www.ncbi.nlm.nih.gov/bioproject/?term=PRJNA1251149, https://www.ncbi.nlm.nih.gov/bioproject/?term=PRJNA1251200, https://www.ncbi.nlm.nih.gov/bioproject/?term=PRJNA1251207, https://www.ncbi.nlm.nih.gov/bioproject/?term=PRJNA1251212, https://www.ncbi.nlm.nih.gov/bioproject/?term=PRJNA1251214, https://www.ncbi.nlm.nih.gov/bioproject/?term=PRJNA1251218, https://www.ncbi.nlm.nih.gov/bioproject/?term=PRJNA1251221 https://www.ncbi.nlm.nih.gov/bioproject/?term=PRJNA1251234, https://www.ncbi.nlm.nih.gov/bioproject/?term=PRJNA1251238 https://www.ncbi.nlm.nih.gov/bioproject/?term=PRJNA1251241, https://www.ncbi.nlm.nih.gov/bioproject/?term=PRJNA1251244 https://www.ncbi.nlm.nih.gov/bioproject/?term=PRJNA1251246, https://www.ncbi.nlm.nih.gov/bioproject/?term=PRJNA1251249, https://www.ncbi.nlm.nih.gov/bioproject/?term=PRJNA1251253, https://www.ncbi.nlm.nih.gov/bioproject/?term=PRJNA1251255, https://www.ncbi.nlm.nih.gov/bioproject/?term=PRJNA1251270, https://www.ncbi.nlm.nih.gov/bioproject/?term=PRJNA1251274, https://www.ncbi.nlm.nih.gov/bioproject/?term=PRJNA1251280 https://www.ncbi.nlm.nih.gov/bioproject/?term=PRJNA1251282, https://www.ncbi.nlm.nih.gov/bioproject/?term=PRJNA1251283, https://www.ncbi.nlm.nih.gov/bioproject/?term=PRJNA1251288, https://www.ncbi.nlm.nih.gov/bioproject/?term=PRJNA1251289].

## Supplementary material

Supplementary Figure 1 Maximum likelihood phylogeny inferred from the core-genome single-nucleotide polymorphisms (SNPs) in 28 genomes identified by Parsnp among the isolates. Branch lengths represent the nucleotide substitutions per site, as indicated by the scale bar.

Supplementary Table 1 Summary of patient conditions.

Supplementary Table 2 Basic genome information on *P. pneumonica* isolates, including genome length and GC content.

Supplementary Table 3 Basic information on potential virulence factor genes, including gene name, genomic location, coverage, similarity, and expression product.

Supplementary Table 4 Basic information on potential resistance genes, including gene name, genomic location, coverage, similarity, and expression product.
