# Supplementary material for: Genomic epidemiology of a novel Pandoraea pneumonica group caused severe bloodstream infection in Hainan, China, 2021-2024
Source: Front Cell Infect Microbiol. 2025 Apr 28;15:1560634. doi: 10.3389/fcimb.2025.1560634 (PMC12066476; doi:10.3389/fcimb.2025.1560634)
Supplement: Supplementary Figure 1 — Maximum likelihood phylogeny inferred from the core-genome single-nucleotide polymorphisms (SNPs) in 28 genomes identified by Parsnp among the isolates. Branch lengths represent the nucleotide substitutions per site, as indicated by the scale bar. [file DataSheet1.zip › Supplementary files/Fig. S1.pdf]

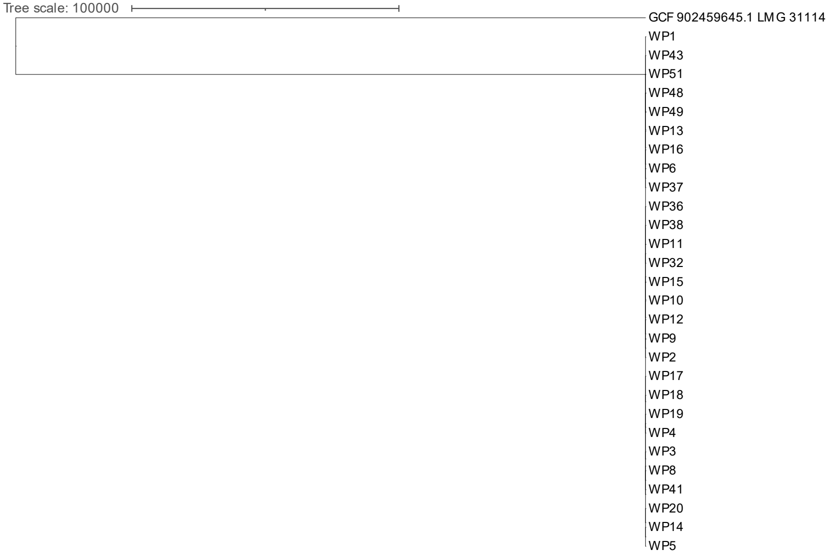
Tree scale: 100000
GCF 902459645.1 LMG 31114
WP1
WP43
WP51
WP48
WP49
WP13
WP16
WP6
WP37
WP36
WP38
WP11
WP32
WP15
WP10
WP12
WP9
WP2
WP17
WP18
WP19
WP4
WP3
WP8
WP41
WP20
WP14
WP5
