# Supplementary material for: Genomic epidemiology of a novel Pandoraea pneumonica group caused severe bloodstream infection in Hainan, China, 2021-2024
Source: Front Cell Infect Microbiol. 2025 Apr 28;15:1560634. doi: 10.3389/fcimb.2025.1560634 (PMC12066476; doi:10.3389/fcimb.2025.1560634)
Supplement: Supplementary Figure 1 — Maximum likelihood phylogeny inferred from the core-genome single-nucleotide polymorphisms (SNPs) in 28 genomes identified by Parsnp among the isolates. Branch lengths represent the nucleotide substitutions per site, as indicated by the scale bar. [file DataSheet1.zip › Supplementary files/HAINAN GENERAL HOSPHAL MEDICAL ETHICS.pdf]

HAINAN GENERAL HOSPHAL MEDICAL ETHICS COMMITTEE'S ETHICS REPORT

Ethical approval No.: Med-Eth-Re[2024]-766

The project, Genomic epidemiology of a novel Pandoraea pneumonica group caused severe bloodstream infection in Hainan, China, 2021-2024 directed by Min Wang form our hospital - was reviewed by the Medical Ethics Committee of Hainan General Hospital. This project conformed to the relevant laws and regulations, and was approved for implementation.

Medical Ethics Committee of Hainan General Hospital

11/25/2024
